# Non-Classical Congenital Adrenal Hyperplasia-Causing Alleles in Adolescent Girls with PCOS and in Risk Group for PCOS Development

**DOI:** 10.3390/diagnostics11060980

**Published:** 2021-05-28

**Authors:** Lasma Lidaka, Laine Bekere, Gunta Lazdane, Iveta Dzivite-Krisane, Anda Kivite-Urtane, Linda Gailite

**Affiliations:** 1 Department of Paediatric Gynaecology, Children’s Clinical University Hospital, LV-1004 Riga, Latvia; 2 Faculty of Medicine, Riga Stradins University, LV-1007 Riga, Latvia; laine.bekere@gmail.com; 3 Department of Obstetrics and Gynaecology, Riga Stradins University, LV-1007 Riga, Latvia; gunta.lazdane@rsu.lv; 4 Department of Public Health and Epidemiology, Public Health Institute of Latvia, Riga Stradins University, LV-1007 Riga, Latvia; anda.kivite-urtane@rsu.lv; 5 Department of Paediatric Endocrinology, Children’s Clinical University Hospital, LV-1004 Riga, Latvia; dzivite@bkus.lv; 6 Scientific Laboratory of Molecular Genetics, Riga Stradins University, LV-1007 Riga, Latvia; linda.gailite@rsu.lv

**Keywords:** PCOS, adolescent, NCAH, alleles, *CYP21A2*

## Abstract

Background: Polycystic ovary syndrome (PCOS) is the most common endocrinopathy in women. Depending on the diagnostic criteria applied, it occurs in up to 16.6% of the general female population. Congenital adrenal hyperplasia includes a group of autosomal recessive disorders, the most common of which is non-classical congenital adrenal hyperplasia (NCAH) caused by mutations in the *CYP21A2* gene. PCOS and NCAH have similar clinical manifestations (hyperandrogenemia, i.e., hirsutism, acne, alopecia, and increased androgen levels in the blood) and potential impact on long-term health (infertility, increased risk of type 2 diabetes, and cardiovascular disease. Consequently, it is thought that NCAH mutations in the heterozygous state may play a role in PCOS development and phenotypic expression. Objective: To determine the prevalence of the most common pathogenic alleles of the *CYP21A2* gene in adolescents with PCOS and adolescents at risk of PCOS development, and to compare the results with healthy adolescents matched for gynecological age. Methods: A cross-sectional study was conducted with 55 PCOS patients, 23 risk patients (with hyperandrogenism but a normal menstrual cycle), and 49 healthy adolescents. Genetic variations in the *CYP21A2* gene were analyzed using a standard Multiplex Ligation-dependent Probe Amplification test (SALSA MLPA Probemix P050-C1 CAH; MRC Holland). Results: No significant differences were found among the three groups regarding the frequency of carriers of NCAH variations in the heterozygous state. It was found that the I172N carrier in the PCOS group had a significantly higher Global Acne Grading Scale score than PCOS patients without this variation (*p* = 0.038). Within the control group of healthy adolescents, compound heterozygous carriers (IVS2-12A > G and -113G > A) had a significantly higher body mass index than non-carriers (*p* = 0.036). Conclusion: We found no differences in the incidence of NCAH-causing variations in the heterozygous state in adolescent PCOS patients, risk adolescents (with hirsutism but normal menstruation), and healthy adolescents. Future studies of larger cohorts and rarer pathogenic *CYP21A2* gene variations are required.

## 1. Introduction

Polycystic ovary syndrome (PCOS) is the most common endocrinopathy in women. Depending on the diagnostic criteria used, its incidence is 4.8 to 16.6% in the general female population [1,2]. PCOS is a multifactorial disease, the development of which involves interaction of both genetic and environmental factors [3]. According to the latest guidelines of the European Society of Human Reproduction and Embryology (ESHRE), a diagnosis can be confirmed in adolescence if two criteria are met: hyperandrogenism (biochemical and/or clinical) and oligomenorrhoea. PCOM on ultrasound is not taken into account in adolescents [4]. Adolescents with signs of PCOS but not meeting all diagnostic criteria are considered a risk population and require additional testing around the gynecological age of 8 years (chronological age minus menarche age), as a full diagnosis of PCOS can only be confirmed over time [4,5].

Congenital adrenal hyperplasia (CAH) includes a group of autosomal recessive disorders associated with enzyme deficiency in the cortisol and aldosterone synthesis pathway [6,7]. In 90% of cases, CAH is caused by a deficiency of the enzyme 21-hydroxylase resulting from genetic mutations in the *CYP21A2* gene on chromosome 6p21.3 [8,9]. The prevalence of non-classical congenital adrenal hyperplasia (NCAH) in the general worldwide population is estimated to be one in 1,000 births [10]. The clinical signs of NCAH in adolescents are similar to those of PCOS, namely hirsutism, acne, irregular menstruation, and PCOM on ultrasound (increased ovarian volume and/or number of secondary follicles) [11,12,13,14,15,16].

At present, the main differential diagnostic tool to exclude the diagnosis of NCAH in patients with hyperandrogenism and menstrual disorders is measurement of the level of 17-OH progesterone in blood on days 3–5 of the menstrual cycle [17]. In a prospective observational study, basal levels of 17-OH progesterone above 2 ng/mL were found in 87% of NCAH patients, 20–25% of PCOS patients, and even 7% of healthy control women [18]. Nonetheless, another prospective study measuring 17-OH progesterone levels following Synacthen administration revealed that heterozygous allele carriers could be missed by solely performing biochemical studies [19]. *CYP21A2* gene variation studies have also been performed in patients with precocious pubarche and complete precocious puberty, which are considered to be antitheses of PCOS. Approximately one-third of these patients (27.7–35.3%) were found to be carriers of *CYP21A2* gene variations in the heterozygous state, compared to 6.0% in healthy adults [20,21,22].

Given the phenotypic similarity between PCOS and NCAH, it has been hypothesized that *CYP21A2* gene variations may be more common in PCOS patients than healthy subjects and consequently affect the phenotype of PCOS patients [23]. However, such studies investigating the frequency of carriers of *CYP21A2* gene variations in the heterozygous state among PCOS patients have provided conflicting results [19,20]. It has also been proposed that a combination of different gene variations could contribute to the development of hyperandrogenism seen in PCOS [24]. The studies published to date have only been conducted in small cohorts, and comparison of their results is difficult due to the different inclusion criteria applied and changes to the PCOS diagnostic criteria over time [4]. In 2018, the ESHRE clarified the diagnostic criteria for the confirmation of PCOS specifically in adolescents [4], thereby helping to select study groups more precisely and, in turn, obtain more reliable results.

The aim of this study was to determine the prevalence of carriers of pathogenic variants of the *CYP21A2* gene in adolescents with PCOS and adolescents at risk of PCOS development and to compare the results with healthy adolescents matched for gynecological age.

## 2. Materials and Methods

For this case-control study, we recruited adolescents between 13 and 18 years of age, at least a year after menarche, who attended the outpatient pediatric gynecology clinic (Children’s Clinical University Hospital, Riga, Latvia). Cases were patients with an established diagnosis of PCOS according to the 2018 criteria of the ESHRE [4]. Control group participants were healthy adolescents who attended the same clinic due to reasons such as contraception counseling or regular health control. Additionally, patients with hirsutism but who did not fulfill all the PCOS criteria and did not have any exclusion criteria were included in the so-called ‘risk’ group.

The exclusion criteria were serious comorbidities (including gynecological and endocrinological (see explanation regarding NCAH exclusion below)) and use of hormonal medication within the previous six months. Recruitment took place between 1 January 2017 and 30 March 2019. The study was approved by the Central Medical Ethics Committee of Latvia (protocol no. 1/16-04-12 and 3/21-02-17). All participants gave their signed informed consent. Permission was also received from legal guardians for those under the age of 16.

The participants underwent a clinical examination and pelvic ultrasound. Reproductive and general medical histories were obtained. Additionally, we assessed their body mass index (BMI), waist–hip ratio, and modified Ferriman–Gallwey (mFG) score to evaluate the degree of hirsutism, Global Acne Grading System (GAGS) score, and menstrual cycle characteristics [4,25]. The levels of total testosterone and 17-OH progesterone were measured in the case group and the risk group patients on days 3–5 of the menstrual cycle (in a certified laboratory using an electrochemiluminescence method (Cobas 6000 immunological analyser; Roche, Basel, Switzerland)). Biochemical hyperandrogenism was defined as an elevated total testosterone level above the normal range on the Tanner scale [4]. In order to exclude participants with NCAH, 17-OH progesterone was evaluated as follows: <2 ng/mL → normal value, NCAH excluded; 2–10 ng/mL → ACTH stimulation test (Synacthen) performed in order to exclude NCAH; >10 ng/mL → referral to endocrinologist, patient not included in the study [17]. Only those subjects who had a normal 17-OH progesterone level or who had a negative Synacthen test were included in the study. Gynecological ultrasound was performed by a single examiner using either an HD11 XE (Philips, Amsterdam, Netherlands) or Logiq P5 (General Electric, Boston, MA, USA) ultrasound machine. A positive finding of PCOM was defined as an ovarian volume >10 mL in at least one ovary and no corpus luteum, dominant follicle, or other cystic structure in any ovary [4]. Ovarian volume was calculated using the simplified formula for a prolate ellipsoid. The larger ovary was used to evaluate ovarian size.

World Health Organization AnthroPlus software was used to calculate BMI and its percentile according to age and normal range for adolescent girls [26]. Weights and heights were measured using standardized calibrated measuring devices.

GENETIC TESTING: A 5 mL sample of peripheral venous blood was obtained from each study participant. DNA was isolated from the samples using a commercial innuPREP DNA Mini Kit (Analytik Jena, Jena, Germany) according to the manufacturer’s protocol. Molecular analysis of the *CYP21A2* gene was performed using a standard Multiplex Ligation-dependent Probe Amplification test (SALSA MLPA Probemix P050-C1 CAH; MRC Holland, Amsterdam, The Netherlands). The probemix included in this MLPA kit contains 33 different probes with amplification products between 130 and 391 bp. This probemix detects several mutations—*CYP21A2* gene deletions, large *CYP21A2/CYP21A1P* gene conversions, a single nucleotide variation 113 bp before the start codon (-113G > A), I2G sequence 13 bp before exon 3 (IVS-12A > C-G), 8-bp deletion in exon 3 (del8bp), I172N variation in exon 4, V237E variation in exon 6, M239K variation in exon 6, and F306 + T variation in exon 7. The MLPA data were analyzed with the Excel-based program Coffalyser 8.0 Software (MRC Holland) [27,28].

STATISTICAL ANALYSIS: IBM SPSS Statistics 22.0 version was used for statistical calculations. The Mann–Whitney U test, Kruskal–Wallis H test, and Fisher’s exact test were used to evaluate the statistical significance of differences of median values or proportions of independent variables among the three study groups.

## 3. Results

The study included 127 adolescents: 55 patients with PCOS, 23 patients in the risk group, and 49 subjects in the healthy control group. The characteristics of the study participants are shown in Table 1. The age of the patients ranged from 12 to 18 years. To characterize the age of participants by group, we chose a more precise characteristic of the adolescent population—gynecological age. Gynecological age did not significantly differ among the groups (*p* = 0.441).

The percentile of BMI was significantly higher in PCOS patients and risk patients compared to control adolescents (*p* < 0.001). However, no significant difference was found between PCOS and risk patients (*p* = 0.320). Furthermore, the waist–hip ratio did not differ significantly between the PCOS patient group and risk patient group (*p* = 0.717), but it was significantly lower in the control group than both these groups (*p* = 0.001).

In the PCOS group, more than one-third of patients (37.3%) had PCOM on ultrasound. PCOM was significantly less common in the risk group and control group (*p* = 0.001).

The GAGS score was significantly higher in the PCOS group than in the risk and control groups (*p* = 0.042 and *p* < 0.001, respectively), but it did not differ significantly between the risk and control groups (*p* = 0.142). The mFG score did not differ significantly between the risk group and PCOS group (*p* = 0.225), but it was significantly higher in both these groups than in the control group (*p* < 0.001).

Altered variants of the CYP21A2 gene were detected in three patients in the PCOS group, two patients in the risk group, and three subjects in the control group (Table 2). None of the study participants carried a variation in the homozygous state. Two of the controls were compound heterozygous carriers of two variations (IVS2-12A > G and -113G > A). The frequencies of allele carriers were not significantly different among the groups; however, the I172N variation was only detected in the PCOS group. Table 3 details the clinical and biochemical characteristics of the eight pathogenic variant carriers.

The relationship between genotype and clinical parameters is shown in Table 4. It was found that the I172N carrier in the PCOS group had a significantly higher GAGS score than PCOS patients without this mutation (*p* = 0.038). Compound heterozygotes (IVS2-12A > G and -113G > A) in the control group had a significantly higher BMI than healthy adolescents without these mutations (*p* = 0.036). A trend-level relationship was observed between the IVS2-12A > G allelic variant and a higher mFG score in risk patients; however, the level of statistical significance was not reached (*p* = 0.091).

## 4. Discussion

The incidences of NCAH variations in the heterozygous state were not significantly different among adolescents with PCOS, risk patients, and healthy controls. It was found that the I172N carrier in the PCOS group had a significantly higher GAGS score than PCOS patients without this mutation (*p* = 0.038). We also observed that compound heterozygous carriers (IVS2-12A > G and -113G > A) in the control group had a significantly higher BMI than healthy adolescents without these mutations (*p* = 0.036).

Our finding that the incidence of carrying NCAH variations in general among PCOS patients is not higher than in control subjects is consistent with the majority of studies published to date. Studying 10 different NCAH-related variations, Escobar-Morreale and colleagues did not find any significant differences in carrier frequency between 40 PCOS patients and 13 healthy controls [29]. Similarly, a study by Witchel and Aston comparing 30 adolescents with PCOS and 14 healthy controls did not reveal any significant differences in the frequency of allele carriers [24]. In contrast, an earlier study by the same group, which included both adolescents with hyperandrogenism and patients with premature pubarche (considered to be a precursor to the development of PCOS), detected a significantly higher incidence of allele carriers in the heterozygous state in both types of patients [20]. Nonetheless, full-sequencing studies of the *CYP21A2* gene failed to expose any significant differences in the frequency of mutation carriers between PCOS patients and healthy controls [24,30].

We found that the I172N carrier in the PCOS group had a significantly higher GAGS score than PCOS patients without this mutation (*p* = 0.038). Although the I172N variation has been included in several studies examining pathogenic *CYP21A2* gene variants in PCOS patients, only one study provided details of allele carriers. Ghanaati and colleagues reported one I172N heterozygous carrier with obesity and oligomenorrhoea, as well as one I172N and IVS2-12A > G compound heterozygous carrier with obesity, hyperandrogenism, and secondary infertility among their PCOS patients. However, a control group was not included in this study, so the contribution of the I172N variation to the PCOS phenotype of these two heterozygous carriers is unclear [31]. In a population of female acne patients (*n* = 51), none of them had the I172N variation [32]. Moreover, a study examining the relationship between *CYP21A2* gene mutations, including the I172N variation, and infertility did not find any significant differences in the frequency of heterozygous carriers between couples with unexplained fertility problems and healthy controls [33].

Compound heterozygous IVS2-12A > G and -113G > A allele carriers in the control group had a significantly higher BMI than healthy adolescents without these mutations (*p* = 0.036). This genotype was not detected in the PCOS group nor the risk group. Both of these variations are in the non-coding region of the gene and cause a severe deficiency of 21-hydroxylase in CAH patients and are associated with severe disease progression [28]. The IVS2-12A > G variation has previously been included in mutation analysis studies investigating the frequency of CAH-determining alleles in PCOS populations; however, there is no information on the contribution of this particular variation to the clinical picture of PCOS. This mutation in the heterozygous state has also been reported in female acne patients, but its association with BMI was not addressed by the authors [32]. This poses a valuable research question for the future.

In this study, we included a variation that has not previously been compared between PCOS patients and healthy controls. This variation, -113G > A, is located in the upstream region of the *CYP21A2* gene where the -113 single nucleotide polymorphism has been replaced by an upstream sequence specific for the *CYP21A2* pseudogene. As a result of the variation, *CYP21A2* transcription is reduced by at least 20% [34]. To date, this variation has been described by Polat and colleagues in a patient with PCOS, along with other promoter region mutations detected by full-gene sequencing [35]. However, control subjects were not included in this study. We did not find a significantly higher incidence of this variation in PCOS or risk patients, nor was it associated with any clinical aspect of PCOS. However, the variation was found in one patient in the risk group and, in combination with IVS2-12A > G, in two subjects in the control group, who in turn had a significantly higher BMI than non-carrier controls. The clinical significance of this allele and its combination requires further investigation, as does the significance of other combinations of gene variations. For example, studies investigating the combination of *CYP21A2* gene variations together with the G972R variant of the insulin receptor substrate-1 gene, which is more common in PCOS patients with severe adrenal hyperandrogenism, have shown interesting results [36].

This study has several strengths. Its data are important because they provide information on the prevalence of pathogenic variants of the *CYP21A2* gene and their association with the phenotype of adolescents with PCOS. This subject matter has rarely been studied in adolescent PCOS patients, while the number of studies in adult PCOS patients is relatively low [31,35,36,37,38]. It should be noted that the comparison of results from different groups is difficult because of varying research methodologies. In our study, PCOS patients were selected using the most recent diagnostic criteria, which were designed specifically for adolescents. Diagnostic criteria have changed significantly over the years, starting with the 1990 NIH criteria [39], then the 2003 Rotterdam criteria [40], and currently the ESHRE 2018 criteria based on rigorous clinical and risk analysis [4]). Therefore, it is very important to universally use the latest diagnostic criteria to build a research knowledge base.

Another strength of our study is the inclusion of a risk group. These adolescents require close monitoring, and genetic testing is essential to understand the development of a particular phenotype and provide a prognosis for the future course of the disease. Our study participants were recruited at the main pediatric gynecology center in the country. This center enables patients from all over the country to be consulted in one place, thus presenting a group of patients representative of the whole country. All the participants forming the three different groups underwent a thorough examination, allowing them to be directly compared with each other. The study examined patients for a wide range of pathogenic variants of the *CYP21A2* gene. The MLPA kit we used detects several variants, including the -113G > A variant, which has not previously been investigated in case-control studies. Most studies analyze all identified pathogenic variants together. However, in our study, the association of each pathogenic variant with the observed clinical features was considered separately, thus providing additional insight into each variant in relation to the clinical picture.

Future studies of larger cohorts would be valuable to confirm the results obtained here and perhaps reveal other possible associations between the *CYP21* genotype and phenotype. Furthermore, although the MLPA kit used in the study allows the detection of a wide range of pathogenic variants of the *CYP21A2* gene, full-gene sequencing would be advantageous for diagnosing subtle changes in the gene. Lastly, extending the analysis to other genes involved in the pathogenesis of PCOS would provide more insight into the genotype–phenotype relationship.

## 5. Conclusions

The frequencies of carriers of heterozygous NCAH variations among PCOS patients, risk patients, and healthy controls were not significantly different in our study sample. Variations were associated with certain clinical characteristics in some patients. Specifically, the I172N variation carrier in the PCOS group had more pronounced acne, and the compound heterozygous IVS2-12A > G and -113G > A allele carriers in the control group had a significantly higher BMI. Future studies of larger cohorts and rarer pathogenic *CYP21A2* gene variations are required to confirm and augment the results of this study.

## Figures and Tables

**Table 1:** Characteristics of the study participants.

Characteristic	PCOS Group (*n* = 55)	Risk Group (*n* = 23)	Control Group (*n* = 49)	*p* Value
Gynecological age, median (IQR)	3.0 (2.0)	4.0 (2.0)	4.0 (1.0)	0.441
mFG score, median (IQR)	9.0 (6.0)	7.5 (6.3)	2.0 (2.0)	**<0.001**
PCOM, *n* (%)	19 (34.5)	4 (17.4)	2 (4.1)	**0.001**
BMI, median percentile (IQR)	89.4 (46.1)	75.6 (38.3)	45.1 (45.8)	**<0.001**
Waist–hip ratio, median (IQR)	0.81 (0.1)	0.81 (0.1)	0.76 (0.1)	**0.001**
GAGS score, median (IQR)	16.0 (17.5)	8.0 (9.0)	6.0 (11.0)	**<0.001**
Testosterone (ng/mL), median (IQR)	0.4 (0.4)	0.3 (0.3)	ND ^1^	0.547
DHEA-SO4 (µg/mL), median (IQR)	221.5 (177.2)	248.0 (190.7)	ND	0.232
Androstenedione (ng/mL), median (IQR)	3.1 (2.7)	2.7 (1.8)	ND	0.287
17-OH progesterone (ng/mL), median (IQR)	1.1 (0.7)	1.3 (1.0)	ND	0.972

^1^ ND—not detected.

**Table 2:** Frequency of identified *CYP21A2* alleles in all study groups.

CYP21A2 Pathogenic Variant	PCOS Group (*n* = 55)	Risk Group (*n* = 23)	Control Group (*n* = 49)	*p* Value
IVS2-12A > G, *n* (%)	2 (3.6)	1 (4.3)	1 (2.0)	0.831
-113G > A, *n* (%)	0 (0)	1 (4.3)	0 (0)	0.181
I172N, *n* (%)	1 (1.8)	0 (0)	0 (0)	1.000
IVS2-12A > G + -113G > A, *n* (%)	0 (0)	0 (0)	2 (4.1)	0.319

**Table 3:** Characterization of each individual with an identified variant(s).

Adolescent	No. 1	No. 2	No. 3	No. 4	No. 5	No. 6	No. 7	No. 8
Group	PCOS group	PCOS group	PCOS group	Risk group	Risk group	Control group	Control group	Control group
CYP21A2 pathogenic variant ^1^	IVS2-12A > G	IVS2-12A > G	I172N	-113G > A	IVS2-12A > G	IVS2-12A > G	IVS2-12A > G + -113G > A	IVS2-12A > G + -113G > A
Age, years	18	17	14	18	16	16	17	16
BMI, percentile	31.7	18.8	31.1	24.6	29.7	22.8	24.5	26.7
Waist–hip ratio	0.93	0.81	0.99	0.82	0.85	0.84	0.69	0.86
mFG score	28	9	8	5	26	2	0	0
Menarche, years	12	13	12	12	12	13	14	14
Menstrual cycle, days	30–90	20–50	90–360	28	28	30	22	21
GAGS score	18	24	31	24	9	13	0	12
PCOM	Detected	Detected	Not detected	Not detected	Not detected	Not detected	Not detected	Not detected
Testosterone, ng/mL	0.59	0.63	0.78	0.64	0.33	NA ^2^	NA	NA
DHEA-SO4, µg/mL	208.0	90.6	180.0	298.0	313.0	NA	NA	NA
Androstenedione, ng/mL	4.10	4.48	5.77	5.88	3.16	NA	NA	NA
17-OH progesterone, ng/mL	1.77	1.45	1.48	1.05	0.99	NA	NA	NA
LH/FSH ratio	1.36	1.62	2.58	0.67	0.35	NA	NA	NA
Glucose, mmol/L	6.11	4.70	4.94	4.65	5.07	NA	NA	NA

^1^ Heterozygous carriers, ^2^ NA—not available.

**Table 4:** Relationship between allelic variants and clinical characteristics.

**Group: PCOS Patients (*n* = 55)**			
**Allelic Variant: I172N**			
**Characteristic**	**Variant Carriers (*n* = 1, 1.8%)**	**Non-Carriers (*n* = 54, 98.2%)**	***p*** **Value**
mFG score, median (IQR)	8	9.0 (6.0)	0.741
PCOM, *n* (%)	0 (0)	19 (35.2)	0.627
BMI, median percentile (IQR)	99.5	89.2 (47.3)	0.353
Waist–hip ratio, median (IQR)	0.99	0.81 (0.14)	0.192
GAGS score, median (IQR)	31	15.5 (16.8)	**0.038**
Testosterone (ng/mL), median (IQR)	0.78	0.40 (0.39)	0.235
**Allelic variant: IVS2-12A > G**			
**Characteristic**	**Variant Carriers (*n* = 2, 3.6%)**	**Non-Carriers (*n* = 53, 96.4%)**	***p*** **Value**
mFG score, median (IQR)	18.5	9.0 (6.0)	0.274
BMI, median percentile (IQR)	59.4	89.4 (45.3)	0.659
Waist–hip ratio, median (IQR)	0.87	0.81 (0.14)	0.462
GAGS score, median (IQR)	21.0	15.0 (17.0)	0.395
Testosterone (ng/mL), median (IQR)	0.61	0.39 (0.40)	0.480
**Group: Risk Patients (*n* = 23)**			
**Allelic Variant: IVS2-12A > G**			
**Characteristic**	**Variant Carriers (*n* = 1, 4.3%)**	**Non-Carriers (*n* = 22, 95.7%)**	***p*** **Value**
mFG score, median (IQR)	26	7.0 (6.0)	0.091
BMI, median percentile (IQR)	98.3	75.4 (39.2)	0.348
Waist–hip ratio, median (IQR)	0.85	0.80 (0.06)	0.273
GAGS score, median (IQR)	9	8.0 (9.5)	0.857
Testosterone (ng/mL), median (IQR)	0.33	0.37 (0.35)	1.000
**Allelic Variant: -113G > A**			
**Characteristic**	**Variant Carriers (*n* = 1, 4.3%)**	**Non-Carriers (*n* = 22, 95.7%)**	***p*** **Value**
mFG score, median (IQR)	5	8.0 (6.0)	0.545
BMI, median percentile (IQR)	82.0	75.4 (40.3)	0.783
Waist–hip ratio, median (IQR)	0.82	0.80 (0.07)	0.910
GAGS score, median (IQR)	24	8.0 (7.8)	0.190
Testosterone (ng/mL), median (IQR)	0.64	0.32 (0.28)	0.471
**Group: Control Subjects (*n* = 49)**			
**Allelic Variant: IVS2-12A > G**			
**Characteristic**	**Variant Carriers (*n* = 1, 2.0%)**	**Non-Carriers (*n* = 48, 98.0%)**	***p*** **Value**
GAGS score, median (IQR)	13	6.0 (10.3)	0.318
BMI, median percentile (IQR)	74.0	45.6 (46.5)	0.429
Waist–hip ratio, median (IQR)	0.84	0.75 (0.06)	0.168
**Allelic Variants: -113G > A + IVS2-12A > G**			
**Characteristic**	**Variant Carriers (*n* = 2, 4.1%)**	**Non-Carriers (*n* = 47, 95.9%)**	***p*** **Value**
GAGS score, median (IQR)	3.5	6.0 (11.5)	0.382
BMI, median percentile (IQR)	89.3	45.1 (45.8)	**0.036**
Waist–hip ratio, median (IQR)	0.77	0.76 (0.06)	0.957

The Mann–Whitney U test, Kruskal–Wallis H test, and Fisher’s exact test were used to evaluate the statistical significance of differences of median values or proportions of independent variables among the three study groups.

## Data Availability

Not applicable.
